# Betanin Modulates the Leptin-Adiponectin Axis and Improves Glycemic Control in a High-Fat Diet and Streptozotocin-Induced Rat Model of Type 2 Diabetes

**DOI:** 10.7759/cureus.104365

**Published:** 2026-02-27

**Authors:** Sagar Agrawal, Rahul Kumar, Ayush Jain, Satyendra Singh, Sarvesh Singh, Rakesh Dixit

**Affiliations:** 1 Pharmacology and Therapeutics, King George's Medical University, Lucknow, IND; 2 Center for Advanced Research, King George's Medical University, Lucknow, IND

**Keywords:** adiponectin, betanin, inflammation, insulin resistance, leptin, type 2 diabetes mellitus

## Abstract

Introduction

Type 2 diabetes mellitus (T2DM) is characterized by persistent hyperglycemia, insulin resistance, and chronic low-grade inflammation, accompanied by adipose tissue dysfunction and altered adipokine secretion. Elevated leptin and reduced adiponectin levels contribute significantly to metabolic deterioration and insulin resistance. Current antidiabetic therapies primarily target glycemic control but inadequately address underlying inflammatory and oxidative mechanisms. Betanin, a natural betalain pigment derived from beetroot, possesses potent antioxidant and anti-inflammatory properties and has shown metabolic benefits in experimental studies. The present study evaluated the effects of betanin on glycemic status and adipokine regulation in a high-fat diet (HFD) and streptozotocin (STZ)-induced rat model of T2DM.

Methodology

Thirty-six adult male Wistar rats were divided into six groups (n = 6): normal control, diabetic control, betanin (10 mg/kg), betanin (20 mg/kg), metformin (100 mg/kg), and metformin plus betanin. Type 2 diabetes was induced by HFD feeding followed by STZ (40 mg/kg). Treatments were administered orally for 28 days. Body weight, fasting blood glucose, and serum leptin and adiponectin levels were assessed. Data were analyzed using one-way ANOVA followed by Tukey’s post-hoc test.

Results

HFD-STZ induction produced significant hyperglycemia, body weight loss, hyperleptinemia, and reduced adiponectin levels compared to normal controls (p < 0.001). Betanin treatment produced dose-dependent improvements, including partial restoration of body weight and a significant reduction in fasting blood glucose. Betanin (20 mg/kg) reduced glucose levels from approximately 302 mg/dL to 188 mg/dL and significantly normalized leptin and adiponectin concentrations (p < 0.001). Metformin showed greater glycemic control and adipokine normalization, while combination therapy did not confer additional benefit over metformin alone.

Conclusion

Betanin significantly ameliorates metabolic dysfunction in an experimental model of T2DM by improving glycemic control and restoring adipokine balance. Its ability to reduce hyperleptinemia and enhance adiponectin secretion supports its potential as a preclinical adjunct strategy targeting metabolic and inflammatory pathways.

## Introduction

Diabetes mellitus is a long-standing metabolic condition marked by sustained elevation of blood glucose levels due to defects in insulin secretion, insulin action, or a combination of both [1]. Its rapidly increasing global prevalence has made it a leading cause of morbidity and mortality. Sustained metabolic imbalance leads to progressive microvascular and macrovascular complications, severely affecting quality of life. These pathological outcomes are driven not only by disturbed glucose metabolism but also by oxidative stress and chronic low-grade inflammation [2].

Type 2 diabetes mellitus develops primarily through insulin resistance in peripheral tissues combined with gradual pancreatic β-cell dysfunction [3]. In early stages, reduced insulin sensitivity in muscle, liver, and adipose tissue necessitates compensatory hyperinsulinemia. Prolonged metabolic stress ultimately overwhelms β-cell capacity, leading to inadequate insulin secretion and overt hyperglycemia [4]. Elevated glucose and circulating lipids promote the generation of reactive oxygen species and the activation of inflammatory signaling pathways, particularly NF-κB and JNK, thereby disrupting insulin receptor signaling and perpetuating metabolic deterioration [2].

Although current therapeutic approaches effectively lower blood glucose, long-term disease control remains suboptimal. Progressive insulin resistance, declining β-cell function, adverse drug effects, and limited impact on inflammatory mechanisms restrict sustained therapeutic success [5]. Importantly, most available antidiabetic agents primarily target glycemia without adequately addressing oxidative stress and chronic inflammation, which are central drivers of disease progression. This highlights the need for adjunct therapies that can simultaneously improve metabolic control and suppress inflammatory pathology [6].

Persistent low-grade systemic inflammation is now recognized as a central contributor to the development and progression of type 2 diabetes. Excess glucose and free fatty acids stimulate pro-inflammatory cascades, leading to increased production of cytokines that interfere with insulin signaling and glucose uptake [7]. Adipose tissue is a major contributor to this inflammatory environment, particularly in obesity, where adipocyte hypertrophy promotes immune cell infiltration and cytokine release [8]. This inflammatory crosstalk between metabolic tissues sustains insulin resistance and accelerates β-cell dysfunction.

Beyond energy storage, adipose tissue functions as an endocrine organ, releasing adipokines that regulate metabolic homeostasis [9]. Leptin and adiponectin are among the most influential adipose-derived hormones in metabolic disease. Elevated leptin levels in obesity and diabetes are associated with leptin resistance and enhanced inflammatory activity, while adiponectin, an insulin-sensitizing and anti-inflammatory hormone, is markedly reduced [10]. The imbalance between increased leptin and decreased adiponectin reflects adipose tissue dysfunction and strongly correlates with insulin resistance and cardiometabolic risk.

Betanin, a naturally occurring betalain pigment derived from beetroot, has gained attention for its potent antioxidant and anti-inflammatory properties [11]. Experimental studies demonstrate that betanin effectively scavenges reactive oxygen species, enhances endogenous antioxidant defenses, and suppresses inflammatory signaling pathways. Additionally, evidence suggests that betanin modulates key metabolic regulators involved in insulin sensitivity and lipid metabolism [12]. In diabetic animal models, betanin administration improves glycemic control, preserves pancreatic architecture, and reduces oxidative and inflammatory damage.

Emerging findings also indicate that betanin may favorably influence adipokine profiles, suggesting a potential role in restoring adipose tissue function [12]. Although preliminary findings are encouraging, detailed assessment of its influence on adipokine regulation in clinically relevant type 2 diabetes models remains scarce.

Experimental animal models are essential for investigating mechanistic pathways underlying metabolic dysfunction, as they allow controlled induction of insulin resistance and β-cell impairment. The high-fat diet (HFD)-streptozotocin (STZ) rat model closely replicates key features of type 2 diabetes, including hyperglycemia, adipokine imbalance, and inflammatory activation. Such controlled preclinical evaluation is necessary before considering translational or clinical investigations [11,12].

Objectives of the study

Therefore, the present study was designed to evaluate the therapeutic effects of betanin in an HFD and STZ-induced rat model of type 2 diabetes. In addition to conventional metabolic parameters, the study focuses on serum leptin and adiponectin as markers of adipose tissue dysfunction and inflammatory status. By integrating biochemical and hormonal assessments, this research aims to clarify whether betanin offers a multidimensional strategy for improving metabolic health and attenuating inflammation in chronic diabetes.

## Materials and methods

Study location and ethical approval

The experimental work was carried out in the Department of Pharmacology at King George’s Medical University, Lucknow, following approval from the Institutional Animal Ethics Committee (IAEC Approval No. 207/IAEC/2024). The study complied with ARRIVE (Animal Research: Reporting of In Vivo Experiments) guidelines for reporting animal research.

Experimental animals

Thirty-six adult male Wistar rats (270-300 g) were obtained from CSIR-Indian Institute of Toxicology Research. Rats were housed in polypropylene cages (six animals per cage) under controlled environmental conditions (temperature 24-26°C, humidity 60-65%, 12-hour light/dark cycle) with free access to standard pellet feed and water. Rats were acclimatized for one week before experimentation. Sample size (n = 6 per group) was selected based on prior comparable experimental studies and ethical reduction principles approved by IAEC.

Induction of type 2 diabetes mellitus

Following acclimatization, rats were divided into six groups (n = 6 per group). The control group received a normal pellet diet (NPD), while the remaining animals were fed an HFD. On day 36, diabetes was induced in HFD-fed rats by a single intraperitoneal injection of STZ (40 mg/kg) [13] freshly prepared in cold sodium citrate buffer (0.1 M, pH 4.5). Rats were maintained on HFD thereafter and allowed to stabilize for one week. Fasting blood glucose was measured after a 12-hour overnight fast (9:00 PM to 9:00 AM), during which animals had free access to water, and those exhibiting values >200 mg/dL [14] were considered diabetic.

Diet composition

NPD and HFD were procured from Om Laboratory & Sales (Lucknow, India). The normal diet provided approximately 3365 kcal/kg, with the majority of energy derived from carbohydrates. The NPD (3365 kcal/kg) consisted of approximately 63% carbohydrates, 18.72% protein, and 4.16% fat. The HFD (5200 kcal/kg) contained 20% carbohydrates, 18.20% protein, and 35.50% fat, providing approximately 60% of total caloric energy from fat [15].

Drugs and chemicals

Metformin was obtained from Cipla (Mumbai, India) and administered orally at 100 mg/kg/day [16]. Betanin was procured from TCI Chemicals (Tokyo, Japan) and given orally at doses of 10 mg/kg/day [12] and 20 mg/kg/day [12]. STZ was sourced from Sisco Research Laboratories Pvt. Ltd. (Mumbai, India).

Experimental design

Animals were allocated to groups using simple randomization following acclimatization and confirmation of eligibility. Each experimental group consisted of six animals (n = 6): Group I: Normal control (NPD + citrate buffer); Group II: Diabetic control (HFD + STZ); Group III: Diabetic + Betanin 10 mg/kg/day; Group IV: Diabetic + Betanin 20 mg/kg/day; Group V: Diabetic + Metformin 100 mg/kg/day; and Group VI: Diabetic + Metformin 100 mg/kg/day + Betanin 20 mg/kg/day.

**Figure 1:**
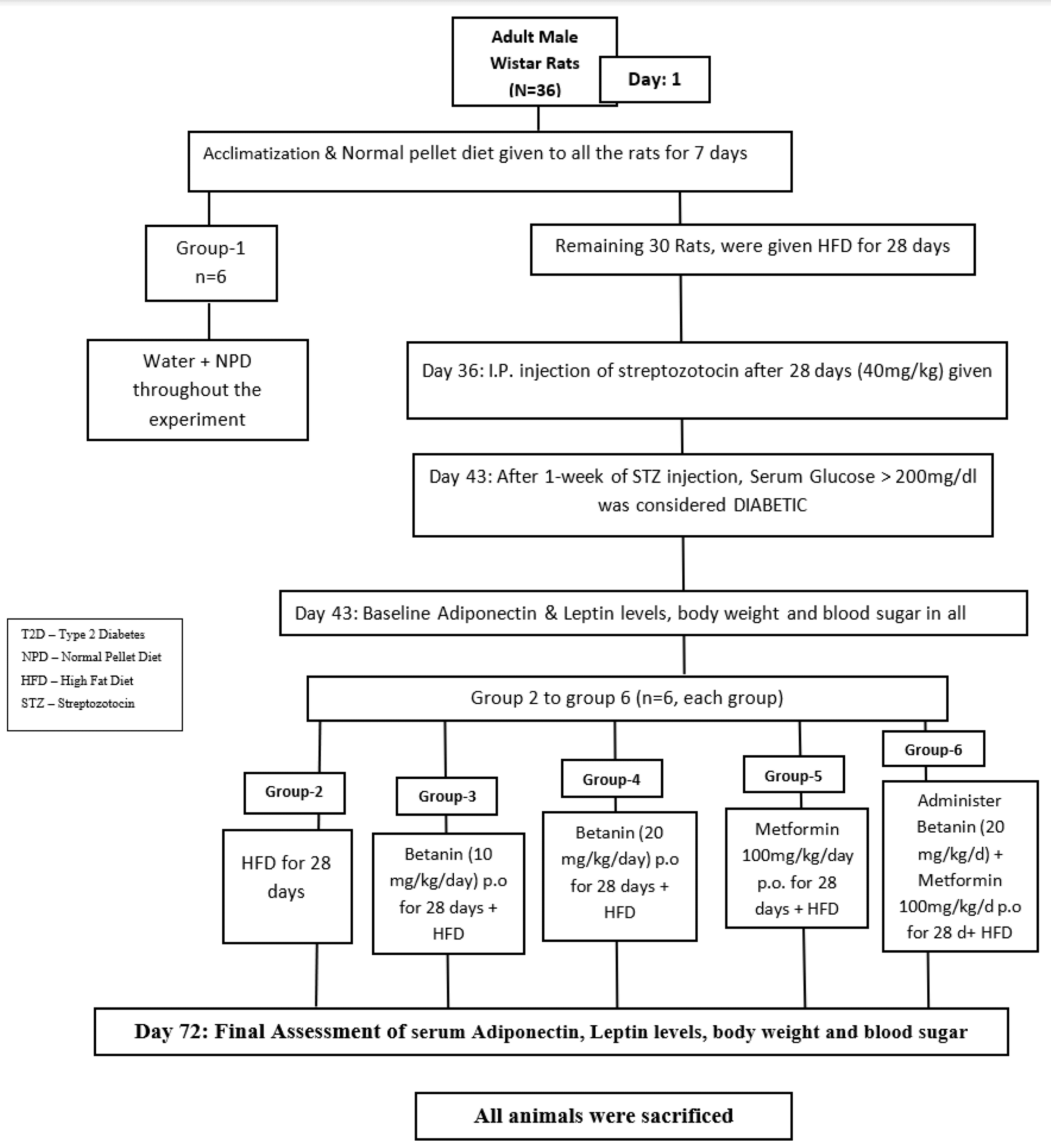
Experimental flow chart

All treatments were administered orally via gavage for 28 consecutive days while maintaining the respective diets. See Figure 1 for the experimental flowchart.

Sample collection and biochemical analysis

Body weight and fasting blood glucose were recorded at baseline and at the end of the treatment period. Blood samples were collected from the tail vein. Serum was separated by centrifugation at 3000 rpm for 20 minutes at 4°C and stored at -80°C until analysis.

Serum leptin and adiponectin concentrations were determined using commercial ELISA kits procured from USCN Business Co., Ltd. (Wuhan, China), following the manufacturer's protocols. Absorbance was read at 450 nm using a microplate reader.

Animal sacrifice

At the conclusion of the experiment, animals were euthanized by intraperitoneal administration of thiopentone sodium (200 mg/kg), resulting in cardiac arrest within 8-10 minutes.

Statistical analysis

Data were expressed as mean ± standard error (SE). Statistical analysis was performed using IBM SPSS Statistics for Windows, Version 24 (Released 2016; IBM Corp., Armonk, New York), one-way analysis of variance (ANOVA) followed by Tukey’s post-hoc test for intergroup comparisons. A p-value < 0.05 was considered statistically significant.

## Results

Body weight

Body weight was recorded at baseline (Day 8), after HFD feeding (Day 36), following STZ administration (Day 43), and at the end of treatment (Day 72). At baseline, all groups exhibited comparable body weights (290-296 g) with no significant intergroup differences (p > 0.05). After 28 days of HFD feeding, rats in Groups II-VI showed a significant increase in body weight compared to the normal control group, confirming successful induction of obesity-associated metabolic stress (p < 0.001).

**Figure 2:**
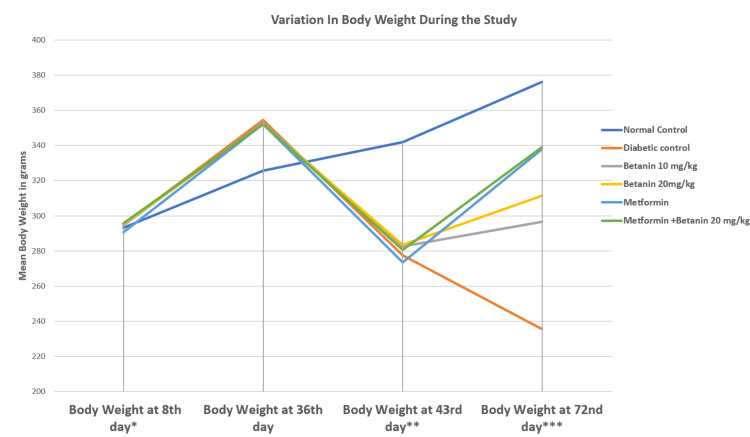
Variation in the body weight during the study period from day 8 to day 72 Values represent mean ± standard error (SE), n = 6 per group. Intergroup comparisons were analyzed using one-way ANOVA followed by Tukey’s post-hoc test. Statistical significance was set at p < 0.05.

Following STZ administration, diabetic groups demonstrated a marked reduction in body weight by Day 43, while normal control animals continued to gain weight. At Day 72, the diabetic control group showed persistent weight loss (p < 0.01). Betanin treatment resulted in a dose-dependent recovery of body weight, with greater improvement observed at 20 mg/kg compared to 10 mg/kg. Metformin-treated rats exhibited substantial restoration of body weight, approaching normal values. Combination therapy produced a comparable effect to metformin alone. Overall, betanin partially attenuated diabetes-associated weight loss, whereas metformin and combination therapy showed superior recovery (Table 1, Figure 2).

**Table 1:** Effect of betanin on body weight in HFD-STZ rats Each group consisted of six animals (n = 6, 100%). Data are expressed as mean ± standard error (SE). Statistical analysis was performed using one-way ANOVA followed by Tukey’s post-hoc test. A p-value < 0.05 was considered statistically significant. *8th Day: Baseline levels, 7 days after acclimatization, ANOVA P-value > 0.05, F-value = 0.38 **43rd Day: Levels 7 days after streptozotocin injection in Groups 2 to 6, ANOVA P-value < 0.01, F-value = 52.79 ***72nd Day: Final levels after treatment, ANOVA P-value < 0.01, F-value = 177.12

Group	Body Weight (in grams) at 8th day (Mean ± SE)*	Body Weight (in grams) at 36th day (Mean ± SE)	Body Weight (in grams) at 43rd day (Mean ± SE)**	Body Weight (in grams) at 72nd day (Mean ± SE)***
1. Normal Control	293.167 ± 2.414	325.667 ± 3.084	342.000 ± 2.436	376.167 ± 2.469
2. Diabetic control	294.833 ± 2.949	354.500 ± 2.432	277.333 ± 4.514	235.500 ± 3.862
3. Betanin 10 mg/kg	294.667 ± 3.116	352.667 ± 2.871	282.500 ± 3.793	296.500 ± 4.924
4. Betanin 20 mg/kg	295.167 ± 2.915	351.833 ± 2.344	283.500 ± 3.658	311.333 ± 2.305
5. Metformin	290.667 ± 3.252	352.167 ± 2.738	273.500 ± 3.019	338.000 ± 2.745
6. Metformin + Betanin 20 mg/kg	295.833 ± 3.400	352.333 ± 2.963	280.500 ± 3.519	339.167 ± 4.468

Blood glucose levels

Fasting blood glucose was assessed at baseline (Day 8), after diabetes induction (Day 43), and at the end of treatment (Day 72). At baseline, all groups exhibited comparable normoglycemic values (88-92 mg/dL) with no significant intergroup differences (p > 0.05). Following STZ administration, all HFD-fed groups developed marked hyperglycemia by Day 43 (≈296-307 mg/dL), confirming successful induction of experimental type 2 diabetes.

**Figure 3:**
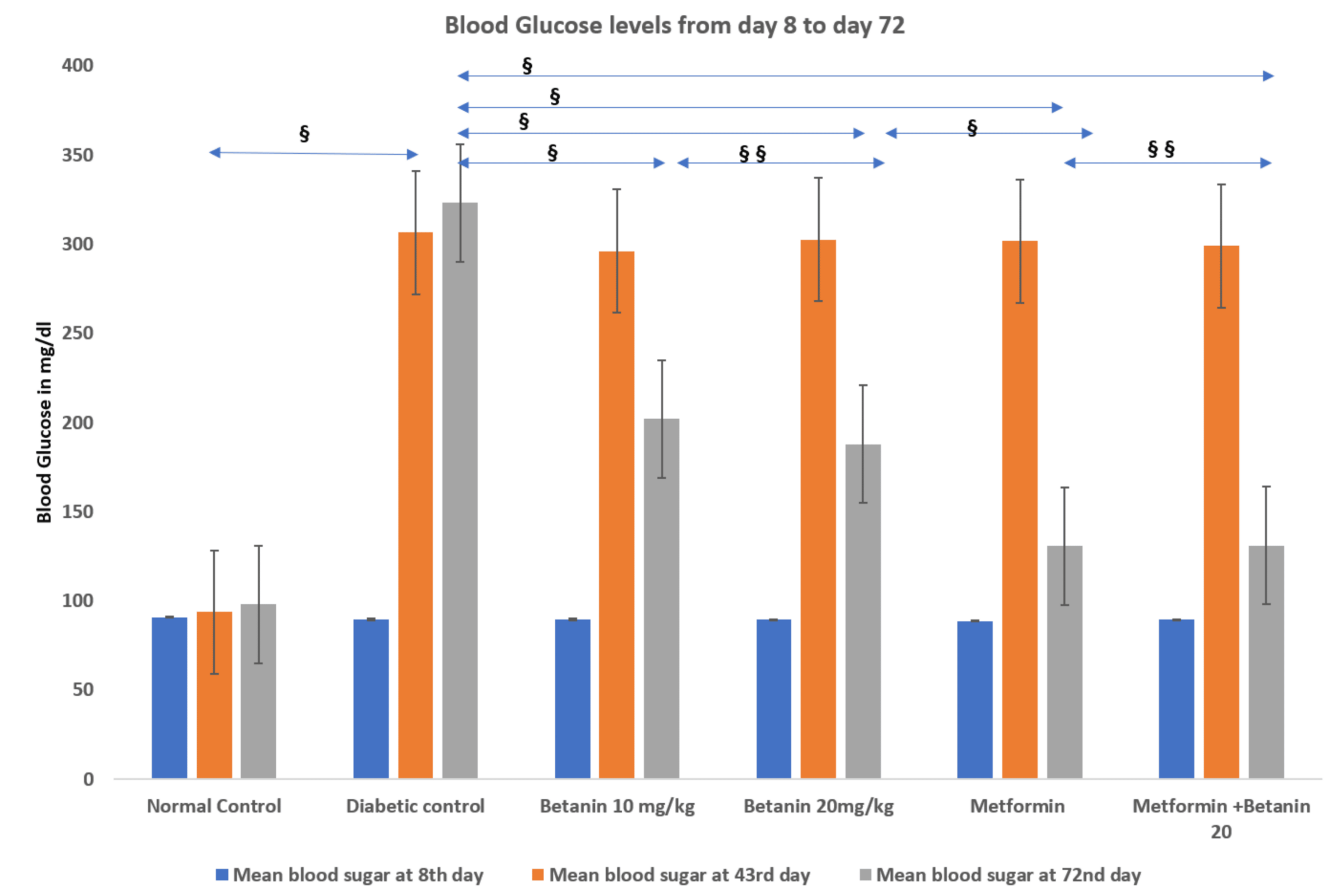
Blood glucose levels on day 8, day 43, and day 72 Values represent mean ± standard error (SE), n = 6 per group. Intergroup comparisons were analyzed using one-way ANOVA followed by Tukey’s post-hoc test. Statistical significance was set at p < 0.05. ^§^P-value < 0.05 ^§§^P-value > 0.05

After 28 days of treatment, the diabetic control group remained severely hyperglycemic. Betanin treatment produced a dose-dependent reduction in fasting blood glucose, with greater improvement observed at 20 mg/kg compared to 10 mg/kg. Metformin treatment resulted in substantial normalization of blood glucose levels. Combined metformin and betanin therapy achieved glycemic control comparable to metformin alone, without additional benefit. Overall, betanin demonstrated moderate antihyperglycemic activity, whereas metformin and combination therapy showed pronounced glucose-lowering efficacy (Table 2, Figure 3).

**Table 2:** Effect of betanin on blood glucose Each group consisted of six animals (n = 6, 100%). Data are expressed as mean ± standard error (SE). Statistical analysis was performed using one-way ANOVA followed by Tukey’s post-hoc test. A p-value < 0.05 was considered statistically significant. *8th Day: Baseline levels, 7 days after acclimatization, ANOVA P-value > 0.05, F-value = 0.38 **43rd Day: Levels 7 days after streptozotocin injection in Groups 2 to 6, ANOVA P-value < 0.01, F-value = 230.14 ***72nd Day: Final levels after treatment, ANOVA P-value < 0.01, F-value = 209.71

Group	Blood Glucose (mg/dL) on the 8th day (Mean ± SE)*	Blood Glucose (mg/d) on the 43rd day (Mean ± SE)**	Blood Glucose (mg/dL) on the 72nd day (Mean ± SE)***
1. Normal Control	91 ± 1.065	93.833 ± 1.167	98.167 ± 1.078
2. Diabetic control	89.667 ± 1.282	306.667 ± 7.265	323.333 ± 10.541
3. Betanin 10 mg/kg	89.667 ± 1.542	296.167 ± 5.63	202.167 ± 5.576
4. Betanin 20 mg/kg	89.5 ± 1.335	302.5 ± 4.958	188 ± 4.531
5. Metformin	88.667 ± 1.145	301.667 ± 6.412	130.833 ± 2.857
6. Metformin + Betanin 20 mg/kg	89.5 ± 0.764	299.167 ± 5.974	131.167 ± 3.798

Serum leptin levels

Serum leptin levels were assessed after diabetes induction (Day 43) and at the end of treatment (Day 72). At Day 43, all diabetic groups exhibited significantly elevated leptin levels (≈3.68-4.13 ng/mL) compared to the normal control group (2.317 ± 0.13 ng/mL), confirming diabetes-associated hyperleptinemia.

By Day 72, the diabetic control group showed a marked increase in leptin levels (15.183 ± 0.338 ng/mL). Betanin treatment significantly reduced leptin concentrations in a dose-dependent manner, with a greater reduction observed at 20 mg/kg (8.9 ± 0.265 ng/mL) compared to 10 mg/kg (12.05 ± 0.349 ng/mL). Metformin treatment resulted in near-normalization of leptin levels (7.017 ± 0.257 ng/mL), while combination therapy produced comparable values (6.85 ± 0.211 ng/mL).

**Figure 4:**
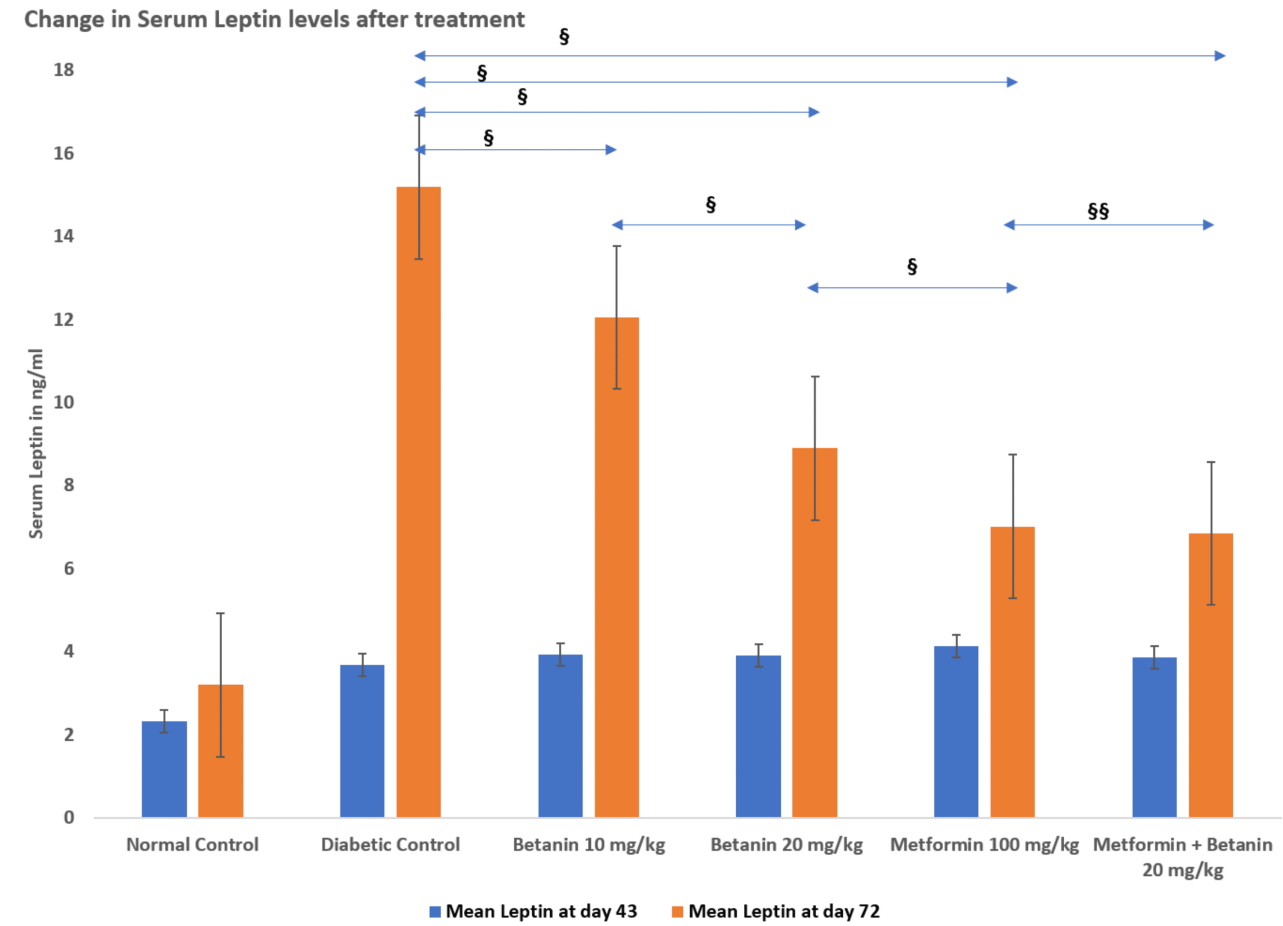
Leptin levels on day 43 and day 72 Values represent mean ± standard error (SE), n = 6 per group. Intergroup comparisons were analyzed using one-way ANOVA followed by Tukey’s post-hoc test. Statistical significance was set at p < 0.05. ^§^P-value < 0.05 ^§§^P-value > 0.05

One-way ANOVA demonstrated a highly significant intergroup difference in leptin levels on Day 72 (F = 239.97, p < 0.01; partial η² = 0.976). Tukey’s post-hoc analysis confirmed that all treatment groups differed significantly from the diabetic control group (p < 0.001). No significant difference was observed between metformin alone and combination therapy (p > 0.05). Overall, betanin significantly attenuated diabetes-associated hyperleptinemia in a dose-dependent manner (Table 3, Figure 4).

**Table 3:** Effect of betanin on serum leptin levels Each group consisted of six animals (n = 6, 100%). Data are expressed as mean ± standard error (SE). Statistical analysis was performed using one-way ANOVA followed by Tukey’s post-hoc test. A p-value < 0.05 was considered statistically significant. *43rd Day: Levels 7 days after streptozotocin injection in Groups 2 to 6, ANOVA P-value < 0.01, F-value = 10.80 **72nd Day: Final levels after treatment, ANOVA P-value < 0.01, F-value = 239.97

Group	Serum Leptin (ng/mL) at day 43 (Mean + SE)*	Serum Leptin (ng/mL) at day 72 (Mean + SE)**
Normal Control	2.317 ± 0.13	3.2 ± 0.177
Diabetic Control	3.683 ± 0.206	15.183 ± 0.338
Betanin 10 mg/kg	3.933 ± 0.236	12.05 ± 0.349
Betanin 20 mg/kg	3.917 ± 0.218	8.9 ± 0.265
Metformin 100 mg/kg	4.133 ± 0.184	7.017 ± 0.257
Metformin + Betanin 20 mg/kg	3.867 ± 0.222	6.85 ± 0.211

Serum adiponectin levels

Serum adiponectin levels were assessed after diabetes induction (Day 43) and at the end of treatment (Day 72). At Day 43, all diabetic groups exhibited significantly reduced adiponectin concentrations (≈3.13-3.27 ng/mL) compared to the normal control group (5.783 ± 0.14 ng/mL), confirming diabetes-associated adipokine dysregulation.

By Day 72, the diabetic control group showed a further decline in adiponectin levels (1.0 ± 0.058 ng/mL). Betanin treatment significantly increased adiponectin concentrations, with comparable improvement observed at 10 mg/kg (4.467 ± 0.187 ng/mL) and 20 mg/kg (4.883 ± 0.158 ng/mL). Metformin treatment resulted in adiponectin levels exceeding normal control values (6.583 ± 0.158 ng/mL), while combination therapy produced a similar increase (6.567 ± 0.186 ng/mL).

**Figure 5:**
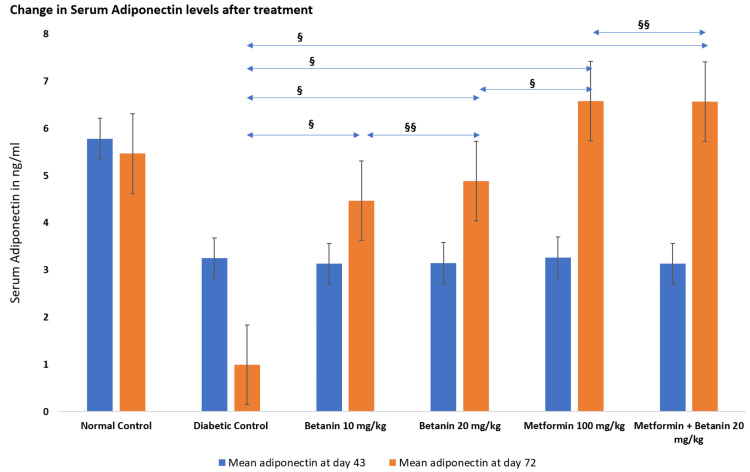
Adiponectin levels at day 43 and day 72 Values represent mean ± standard error (SE), n = 6 per group. Intergroup comparisons were analyzed using one-way ANOVA followed by Tukey’s post-hoc test. Statistical significance was set at p < 0.05. ^§^P-value < 0.05 ^§§^P-value > 0.05

One-way ANOVA revealed highly significant intergroup differences in adiponectin levels on Day 72 (F = 196.77, p < 0.01; partial η² = 0.970). Tukey’s post-hoc analysis showed that all treatment groups differed significantly from the diabetic control group (p < 0.001), with no significant difference between betanin doses or between metformin alone and combination therapy (p > 0.05). Overall, betanin significantly reversed diabetes-induced adiponectin suppression, while metformin and combination therapy produced robust restoration (Table 4, Figure 5).

**Table 4:** Effect of betanin on serum adiponectin levels Each group consisted of six animals (n = 6, 100%). Data are expressed as mean ± standard error (SE). Statistical analysis was performed using one-way ANOVA followed by Tukey’s post-hoc test. A p-value < 0.05 was considered statistically significant. *43rd Day: Levels 7 days after streptozotocin injection in Groups 2 to 6, ANOVA P-value < 0.01, F-value = 51.26 **72nd Day: Final levels after treatment, ANOVA P-value < 0.01, F-value = 196.77

Group	Serum Adiponectin (ng/mL) at day 43 (Mean + SE)*	Serum Adiponectin (ng/mL) at day 72 (Mean + SE)**
Normal Control	5.783 ± 0.14	5.467 ± 0.084
Diabetic Control	3.25 ± 0.177	1 ± 0.058
Betanin 10 mg/kg	3.133 ± 0.145	4.467 ± 0.187
Betanin 20 mg/kg	3.15 ± 0.099	4.883 ± 0.158
Metformin 100 mg/kg	3.267 ± 0.133	6.583 ± 0.158
Metformin + Betanin 20 mg/kg	3.133 ± 0.18	6.567 ± 0.186

## Discussion

The present study demonstrates that betanin exerts significant metabolic and anti-inflammatory effects in an HFD and STZ-induced model of type 2 diabetes mellitus. Induction of diabetes resulted in obesity-associated metabolic derangements followed by severe hyperglycemia, weight loss, hyperleptinemia, and suppression of adiponectin, reflecting classical features of insulin resistance, adipose tissue dysfunction, and chronic inflammation [7,15].

Treatment with betanin significantly attenuated these pathological changes in a dose-dependent manner, indicating its potential as a multifunctional anti-diabetic agent. Previous studies show a similar pattern [11,12].

HFD feeding produced marked weight gain before STZ administration, confirming the successful induction of obesity-associated insulin resistance. This was followed by rapid body weight loss after STZ injection, consistent with β-cell injury, impaired glucose utilization, and enhanced protein and lipid catabolism typical of uncontrolled diabetes. Persistent weight loss in diabetic control animals further reflects metabolic decompensation. Betanin treatment significantly improved body weight recovery, particularly at the higher dose, suggesting restoration of metabolic efficiency and improved insulin action. This recovery likely results from enhanced glucose uptake, reduced oxidative tissue injury, and improved adipose endocrine function [17].

STZ-induced diabetes produced pronounced hyperglycemia, confirming β-cell dysfunction superimposed on insulin resistance. Betanin administration reduced fasting blood glucose in a dose-dependent manner, although not to the extent observed with metformin. This moderate but significant antihyperglycemic effect suggests that betanin primarily improves insulin sensitivity and cellular glucose utilization rather than directly stimulating insulin secretion. Activation of metabolic regulators such as AMPK, suppression of hepatic gluconeogenesis, and reduction of oxidative stress likely contribute to improved glycemic control [3,18].

A key finding of the present study is the profound alteration in adipokine profiles induced by diabetes and their restoration following betanin therapy. Diabetic rats exhibited elevated leptin levels, indicating leptin resistance and adipose tissue inflammation. Hyperleptinemia is known to amplify inflammatory signaling through activation of NF-κB and cytokine production, further worsening insulin resistance. The marked increase in leptin observed in diabetic controls reflects progressive adipose dysfunction and systemic inflammation [12,19].

Betanin significantly reduced leptin levels in a dose-dependent manner, suggesting suppression of inflammatory adipokine overproduction and possible improvement in leptin sensitivity. This effect is consistent with betanin’s ability to inhibit inflammatory transcription pathways and oxidative stress. Lower leptin levels following treatment indicate reduced adipose tissue inflammation and improved metabolic signaling [20].

Conversely, adiponectin levels were markedly suppressed following diabetes induction, reflecting loss of insulin-sensitizing and anti-inflammatory protection. Adiponectin plays a crucial role in enhancing glucose uptake, fatty acid oxidation, and suppressing pro-inflammatory cytokine production. Its decline in diabetes contributes directly to worsening insulin resistance and metabolic inflammation [21].

Betanin treatment significantly restored adiponectin levels, with higher doses producing greater improvement. This restoration likely contributes to enhanced insulin sensitivity, improved lipid metabolism, and suppression of inflammatory signaling. The increase in adiponectin further supports activation of AMPK-mediated metabolic pathways and improved mitochondrial function. Metformin and combination therapy produced even higher adiponectin levels, consistent with their strong insulin-sensitizing actions [16].

The combined modulation of leptin and adiponectin by betanin represents a central mechanistic pathway through which metabolic and inflammatory balance is restored. Reduction of leptin-driven inflammation alongside enhancement of adiponectin-mediated insulin sensitivity breaks the vicious cycle linking obesity, inflammation, and hyperglycemia. This dual adipokine correction provides mechanistic support for the observed improvements in body weight and blood glucose.

The strong statistical effects observed for both leptin and adiponectin indicate that betanin produces biologically meaningful endocrine remodeling of adipose tissue rather than isolated biochemical changes. These findings align with emerging evidence that targeting inflammatory-metabolic crosstalk is essential for long-term diabetes control.

While metformin demonstrated superior glucose-lowering efficacy, betanin offered substantial anti-inflammatory and adipokine-modulating benefits. The combination therapy did not exhibit marked synergistic superiority over metformin alone, suggesting overlapping insulin-sensitizing pathways. However, betanin’s antioxidant and cytokine-suppressing actions may offer protective benefits not fully achieved by conventional therapies.

**Figure 6:**
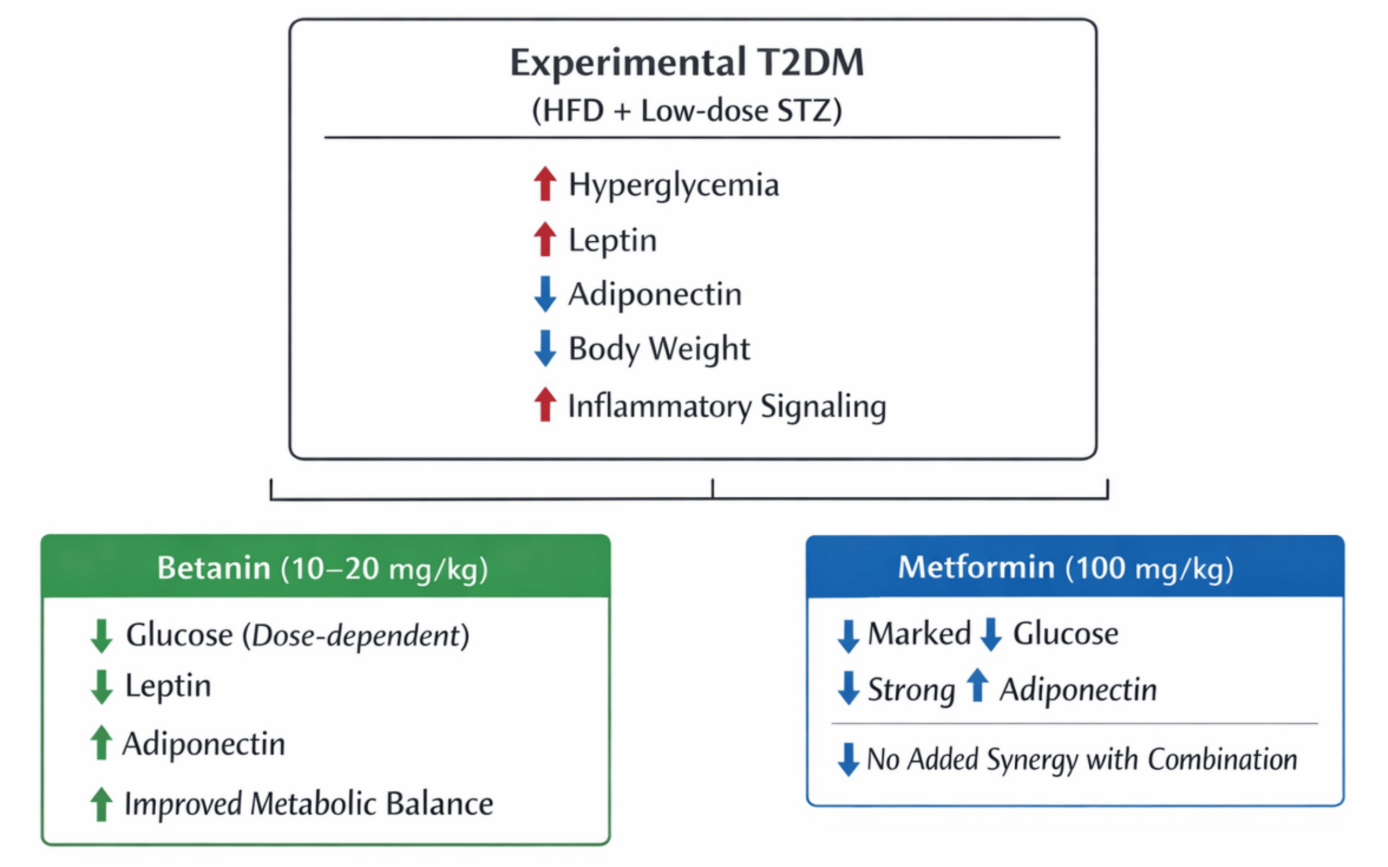
Betanin modulates the leptin-adiponectin axis in an experimental model of type 2 diabetes mellitus Schematic representation of metabolic alterations induced by high-fat diet and low-dose streptozotocin (HFD-STZ) and the modulatory effects of betanin and metformin. Experimental diabetes was characterized by hyperglycemia, elevated leptin levels, reduced adiponectin concentrations, body weight loss, and increased inflammatory signaling. Betanin treatment (10–20 mg/kg) improved glycemic control and restored adipokine balance in a dose-dependent manner, while metformin (100 mg/kg) demonstrated stronger glucose-lowering efficacy and marked adiponectin restoration. Combination therapy did not show additional synergistic benefit. Restoration of the leptin-adiponectin axis is proposed as a central mechanism underlying improved insulin sensitivity and reduced inflammatory burden in this experimental model. Image created by the authors using Microsoft PowerPoint (Microsoft Corporation, Redmond, Washington).

Overall, the present study supports the concept that diabetes progression is driven not only by hyperglycemia but also by adipose tissue inflammation and adipokine imbalance. Betanin effectively targets these underlying mechanisms by reducing oxidative stress, suppressing inflammatory signaling, lowering leptin overproduction, and restoring adiponectin secretion. These coordinated actions improve insulin sensitivity, metabolic stability, and endocrine function (Figure 6).

Limitations of the study

Despite the promising findings, several limitations of the present study should be acknowledged. First, the investigation was conducted in an experimental animal model, which, although closely mimicking human type 2 diabetes pathophysiology, may not fully replicate the complexity of metabolic regulation and disease progression observed in human patients. Therefore, direct extrapolation of these results to clinical settings should be approached with caution.

Second, only male rats were included, which may limit generalizability due to potential sex-related differences in metabolic and adipokine regulation.

Third, the study primarily focused on biochemical and hormonal parameters without detailed molecular analysis of intracellular signaling pathways. In addition, serum insulin levels were not directly measured; thus, insulin resistance indices were not assessed. Direct evaluation of key molecular targets such as AMPK, NF-κB, insulin receptor substrates, oxidative stress markers, and circulating insulin would provide deeper mechanistic insight.

Fourth, the treatment duration was limited to 28 days, which may not adequately reflect the long-term efficacy and safety of betanin administration in chronic diabetes. Extended studies are required to determine sustained metabolic benefits and potential cumulative effects.

Finally, although combination therapy with metformin was explored, potential pharmacodynamic interactions were not extensively investigated. Further research is warranted to clarify synergistic or antagonistic effects when betanin is used alongside standard antidiabetic drugs.

Future directions of the study

Future studies should investigate the molecular mechanisms underlying betanin’s metabolic effects, particularly its interaction with AMPK, NF-κB, insulin receptor signaling pathways, and oxidative stress mediators. Evaluation of serum insulin levels and insulin resistance indices (e.g., HOMA-IR), inclusion of female subjects to assess sex-specific responses, and long-term efficacy and safety studies are warranted. Ultimately, well-designed clinical trials will be necessary to determine the translational relevance of betanin as a complementary strategy in type 2 diabetes management.

## Conclusions

The present study demonstrates that betanin confers significant metabolic and anti-inflammatory benefits in an HFD and STZ-induced rat model of type 2 diabetes mellitus. Experimental diabetes was characterized by hyperglycemia, weight loss, elevated leptin, and reduced adiponectin levels, indicating insulin resistance and adipose tissue dysfunction. Betanin treatment produced dose-dependent improvements in glycemic control, partially restored body weight, reduced hyperleptinemia, and enhanced adiponectin levels, suggesting modulation of the leptin-adiponectin axis as a key underlying mechanism.

Although metformin exhibited stronger antihyperglycemic effects, betanin demonstrated notable adipokine-regulating and anti-inflammatory properties, supporting its potential as a complementary therapeutic agent. Overall, these findings indicate that betanin targets both metabolic and inflammatory components of type 2 diabetes and merits further investigation to establish its translational relevance.
